# Effect of Different Cavity Disinfectants on Adhesion to Dentin of Permanent Teeth

**DOI:** 10.3390/jfb13040209

**Published:** 2022-10-28

**Authors:** Ana Coelho, Luís Vilhena, Maria Antunes, Inês Amaro, Anabela Paula, Carlos Miguel Marto, José Saraiva, Manuel Marques Ferreira, Eunice Carrilho, Amílcar Ramalho

**Affiliations:** 1 Institute of Integrated Clinical Practice, Faculty of Medicine, University of Coimbra, 3000-075 Coimbra, Portugal; 2 Area of Environment Genetics and Oncobiology (CIMAGO), Faculty of Medicine, Coimbra Institute for Clinical and Biomedical Research (iCBR), University of Coimbra, 3000-548 Coimbra, Portugal; 3 Clinical Academic Center of Coimbra (CACC), 3004-561 Coimbra, Portugal; 4 Department of Mechanical Engineering, Centre for Mechanical Engineering, Materials and Processes (CEMMPRE), University of Coimbra, 3004-516 Coimbra, Portugal; 5 Institute of Biophysics, Faculty of Medicine, University of Coimbra, 3004-548 Coimbra, Portugal; 6 Institute of Experimental Pathology, Faculty of Medicine, University of Coimbra, 3004-548 Coimbra, Portugal; 7 Institute of Endodontics, Faculty of Medicine, University of Coimbra, 3000-075 Coimbra, Portugal

**Keywords:** adhesion, cavity disinfectants, dental caries, shear bond strength

## Abstract

After the elimination of dental caries lesions, some microorganisms may remain viable in the tooth structure. Thus, cavity disinfection is an important procedure. The aim of this study was to evaluate the effect of cavity disinfectants on the adhesion to dentin of permanent teeth. Sixty molars were ground flat and randomly assigned to six groups: control; chlorhexidine; Aloe vera; glutaraldehyde; EDTA; ethanol. Cavity disinfectants were applied, rinsed, and air-dried. The restorations were performed with the aid of polyethylene tubes. Shear bond strength, work to detachment, and shear modulus were evaluated. All data were statistically analyzed and the level of significance was set at 5%. The control group showed the lowest shear bond strength (8.34 ± 2.68 MPa). Aloe vera showed the lowest work to debonding (2284 J/m^2^) while chlorhexidine showed the highest (9347 J/m^2^). Regarding the shear modulus, ethanol, chlorhexidine, and EDTA presented similar values to the control group (216.11 kPa), and glutaraldehyde and Aloe vera presented values twice as high. The use of chlorhexidine, ethanol, EDTA, glutaraldehyde, and Aloe vera did not impair the adhesion established between the dentin of permanent teeth and composite resin. Even though there is a need for clinical studies to support these findings, all disinfectants seem to be good choices as pretreatment agents.

## 1. Introduction

Dental caries is one of the most prevalent diseases in the world. Its progression results from an imbalance of the continuous cycle of demineralization and remineralization of the tooth structure, resulting from a complex interaction between acidogenic bacteria (namely *Streptococcus mutans* and *Lactobacillus* spp.), fermentable carbohydrates, and host factors [1,2,3,4,5].

Treatment of dental caries consists of the elimination of the carious and necrotic tissue and subsequent rehabilitation of the remaining structure [1]. However, some microorganisms may remain viable in the tooth structure, which may compromise the success of the rehabilitation by leading to the development of secondary caries lesions, increased dentin sensitivity, and/or pulp inflammation [6].

Thus, cavity disinfection is an important procedure to be performed before the restoration of the remaining structure [7,8].

Over the past few years, several products have been proposed to be used as cavity disinfectants. However, the effect of applying cavity disinfectants on adhesion is not yet clear for most of the proposed products [7,9,10]. On the other hand, contrary to what happens with enamel, adhesion to dentin is a technical challenge, given the inherent humidity of the substrate and its heterogeneity. As so, the effect of applying a cavity disinfectant needs to be evaluated [11].

The aim of the present study was to evaluate the effect of different cavity disinfectants on the adhesion of the composite resin to the dentin of permanent teeth.

Considering the results of a recent systematic review [10] on the topic, a control group (no cavity disinfectant application) was established, chlorhexidine was chosen as the gold standard, and ethanol, EDTA, Gluma^®^ (Heraeus, Germany), and Aloe vera were selected as tests groups since there is a lack of evidence regarding the effect of these disinfectant agents on adhesion to the dentin of permanent teeth.

The established null hypothesis was that there were no significant differences between disinfectants and control.

## 2. Materials and Methods

All data relating to the patients included in the study were kept confidential and anonymized, in accordance with the applicable regulations and laws, as well as the Declaration of Helsinki and its updates, after being approved by the Ethics Committee of the Faculty of Medicine of the University of Coimbra (CE-096-2021).

The study included 60 intact third molars extracted due to pericoronitis, impaction, inclusion, or orthodontic reasons, from patients of the Dentistry Area of the Faculty of Medicine of the University of Coimbra. All participants read, understood, and signed an informed consent form.

Teeth with caries, restorations, structural defects, erosion, or abfraction, as well as teeth that fractured during the extraction were excluded.

After extraction, all teeth were stored in distilled water at 4 °C for a maximum period of one month, according to ISO/TS 11405:2015 (Dental materials—testing of adhesion to tooth structure) [12].

Occlusal thirds of the molars were ground flat using a diamond disk (918BF/220, D+Z, Germany) under running water to provide dentin surfaces perpendicular to the long axis of the teeth. The sections of the teeth including the roots were embedded in auto-polymerizing acrylic resin (Probase, Ivoclar Vivadent, Spain) using a polyvinyl chloride (PVC) cylinder 2.5 cm in diameter and 5 cm high.

The cavity disinfectants were applied according to the following groups:

Group 1—control—no application of cavity disinfectants (n = 10);

Group 2—disinfection with 0.20% chlorhexidine—Eludril Extra (Pierre Fabre, France) (n = 10);

Group 3—disinfection with Aloe vera (Just Jaivikâ, Herbs, and Crops Overseas, India) (n = 10);

Group 4—disinfection with 5% glutaraldehyde (Gluma^®^, Heraeus, Germany) (n = 10);

Group 5—disinfection with 17% ethylenediamine tetraacetic acid (EDTA, EDTA PLUS^®^, Clarben, Lithuania) (n = 10);

Group 6—disinfection with 100% ethanol (Sigma-Aldrich Corporation) (n = 10).

Table 1 presents the components and manufacturers of the disinfectants used in the study.

Aloe vera solution was prepared using *Aloe barbadensis* powder 200:1 concentrate (Just Jaivikâ, Herbs and Crops Overseas, India) and dissolving 0.5 g of Aloe vera powder in 99.5 g of distilled water.

Cavity disinfectants were actively applied with a microbrush for 30 s and rinsed for 30 s. The dentin surfaces of the teeth were then air-dried for 15 s and the bonding agent (Scotchbond Universal, 3M, USA) was actively applied with a microbrush for 20 s and light cured for 40 s using the Smart Lite Focus unit (Dentsply Sirona, Charlotte, North Carolina, USA).

Resin composite (Admira Fusion, Voco, Germany) was applied in 2–3 increments with the aid of polyethylene tubes (3 mm diameter, 2 mm height) and light cured for 40 s.

**Figure 1:**
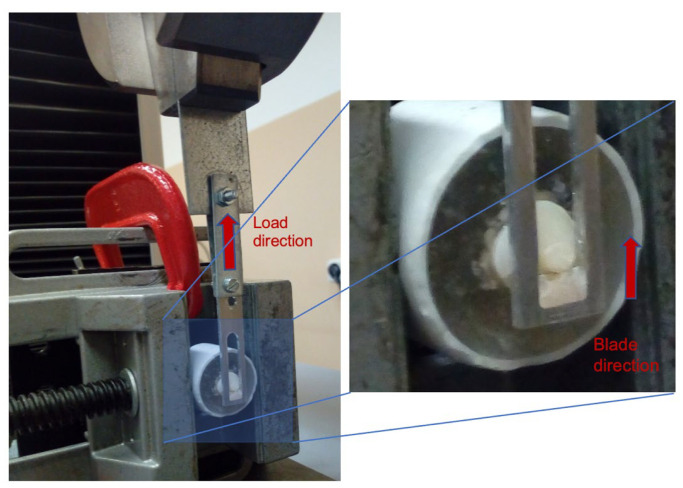
Assembly used to determine the shear bond strength between dentin and composite resin in a Shimadzu universal testing machine.

Shear bond strength (SBS), work to detachment (WD), and shear modulus (SM) were evaluated in vitro for the different prepared specimens according to ISO 29022 (2013) and DIN 13990 (2017) standards. The equipment used was a Shimadzu universal testing machine (Shimadzu Autograph AG-X-5kN, Kyoto, Japan), equipped with a 5 kN load cell, as exemplified in Figure 1. The direction of the application of the force was parallel to the interface between the dentin surface and the composite resin, using a blade with a guillotine window to incorporate the composite resin cylinder until detachment occurs. The travel speed was 0.5 mm/min. To analyze the morphology, the dentin surface of one specimen from each group was observed before and after mechanical testing with a Hitachi SU-3800 Scanning Electron Microscope (Hitachi High Technologies, Tokyo, Japan).

**Figure 2:**
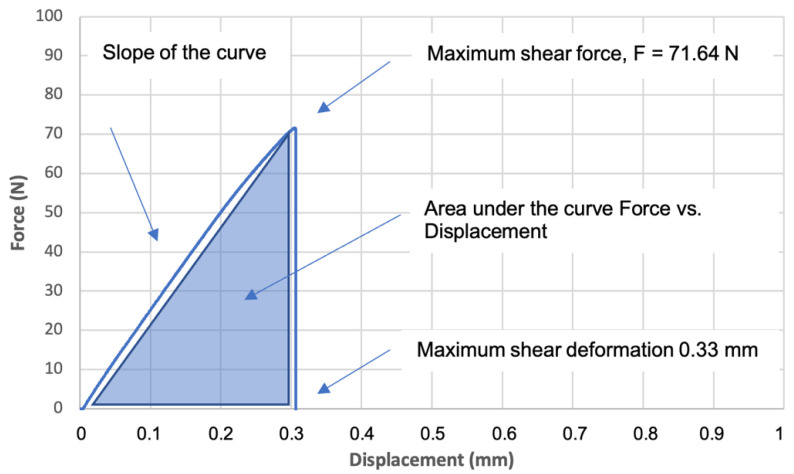
Example of shear test performed for group 3—disinfection with Aloe vera, specimen Al5.

Through the shear tests with the adhesive joint, as exemplified in Figure 1, it was possible to plot the force vs. displacement curve, as shown in Figure 2, which allowed us to analyze the behavior of the adhesive joint and to determine different mechanical properties, such as maximum shear strength, maximum shear deformation, work to detachment, and shear modulus. The maximum shear strength was determined as the load at the detachment point normalized by the adhesive area (MPa). The work to detachment gives the ability of a given adhesive joint to absorb energy and indicates the durability of the adhesive joint that is subjected to repetitive stresses. This parameter was determined by calculating the area under the curve force vs. displacement normalized by the adhesive area, as shown in Figure 2 and presented in J/m^2^. The shear modulus or rigidity modulus was calculated based on the slope of the force vs. displacement curve, normalized by the area and the thickness of the adhesive area, and is given in kPa.

Results were presented as mean ± standard error of the mean. All data were analyzed using Kruskal–Wallis and Dunn’s post hoc tests. The level of significance assumed was 5%. The software used in data processing was IBM^®^ SPSS^®^ v.27.0 (IBM Corporation, USA).

## 3. Results

**Figure 3:**
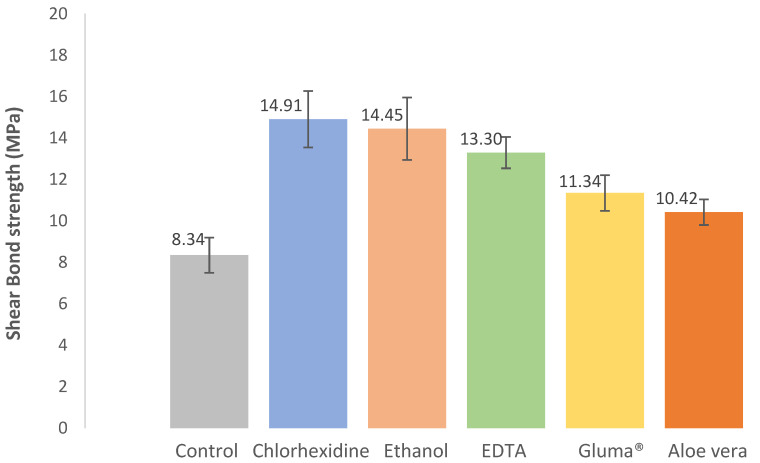
Effect of cavity disinfectants on shear bond strength. Error bars represent standard errors of the mean.

The results regarding the shear bond strength are shown in Figure 3. It is possible to observe that the control group showed the lowest shear bond strength of the order of 8.34 ± 0.85 MPa, while all the other groups showed higher shear bond strengths between 10.42 ± 0.62 MPa and 14.91 ± 1.36 MPa, corresponding in percentage terms to an increase between 24.9 and 78.7%, respectively.

Statistically, there was a significant difference in shear bond strength between the different groups (X^2^(5) = 22.995, *p* < 0.001). There were significant differences between control and chlorhexidine (*p* = 0.003), control and ethanol (*p* = 0.008), and control and EDTA (*p* = 0.009), demonstrating that these groups performed better than the control group, using only the bonding agent without any cavity disinfectant.

**Figure 4:**
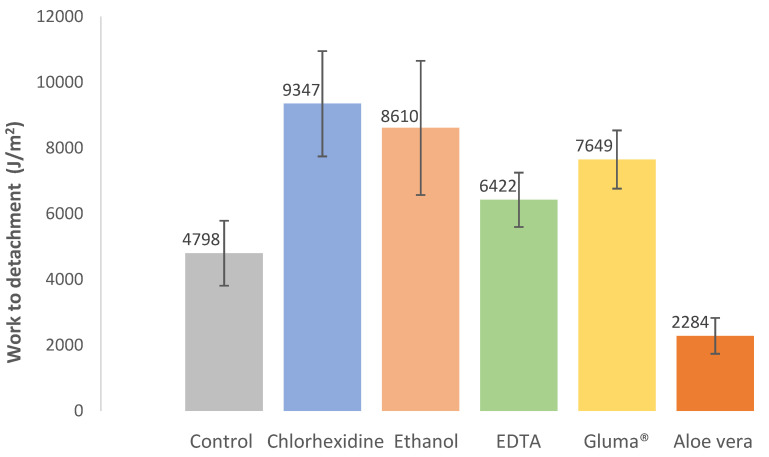
Effect of cavity disinfectants on work to detachment. Error bars represent standard errors of the mean.

The results regarding the work to detachment are shown in Figure 4. This parameter indicates the ability of a given adhesive joint to absorb energy without debonding or detachment. As can be seen by analyzing Figure 4, the Aloe vera group showed the lowest work to debonding of the order of 2284 J/m^2^, while the chlorhexidine group showed the highest with 9347 J/m^2^.

Statistically, there was a significant difference in work to detachment between the different groups (X^2^(5) = 16.685, *p* = 0.005). There were significant differences between control and chlorhexidine (*p* = 0.011) and control and Gluma^®^ (*p* = 0.015).

**Figure 5:**
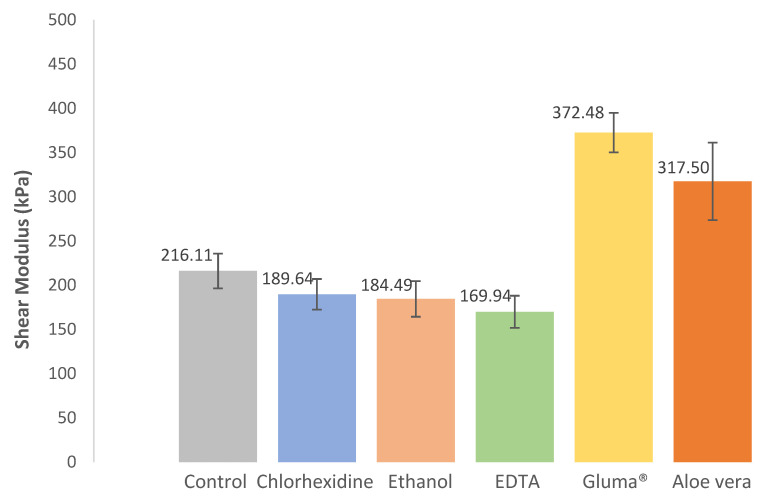
Effect of cavity disinfectants on the shear modulus or rigidity modulus. Error bars represent standard errors of the mean.

The results regarding the shear modulus or modulus of rigidity are shown in Figure 5. By analyzing Figure 5, it can be seen that there were essentially two distinct groups: one that presented similar values to the control group (216.11 kPa), composed of the ethanol, chlorhexidine, and EDTA groups with values varying between 169.94 kPa and 189.64 kPa, and the other that presented values twice as high of the control group, composed of the Gluma^®^ and Aloe vera groups, with values varying between 317.50 kPa and 372.48 kPa.

Statistically, there was a significant difference in the rigidity modulus between the different groups (X^2^(5) = 15.100, *p* < 0.001). There were significant differences between Aloe vera and EDTA (*p* = 0.018), Gluma^®^ and EDTA (*p* < 0.001), Gluma^®^ and ethanol (*p* = 0.003), Gluma^®^ and chlorhexidine (*p* = 0.007), and Gluma^®^ and control (*p* = 0.043).

All the results regarding shear bond strength, shear modulus, and work to detachment can be found in Table 2.

**Figure 6:**
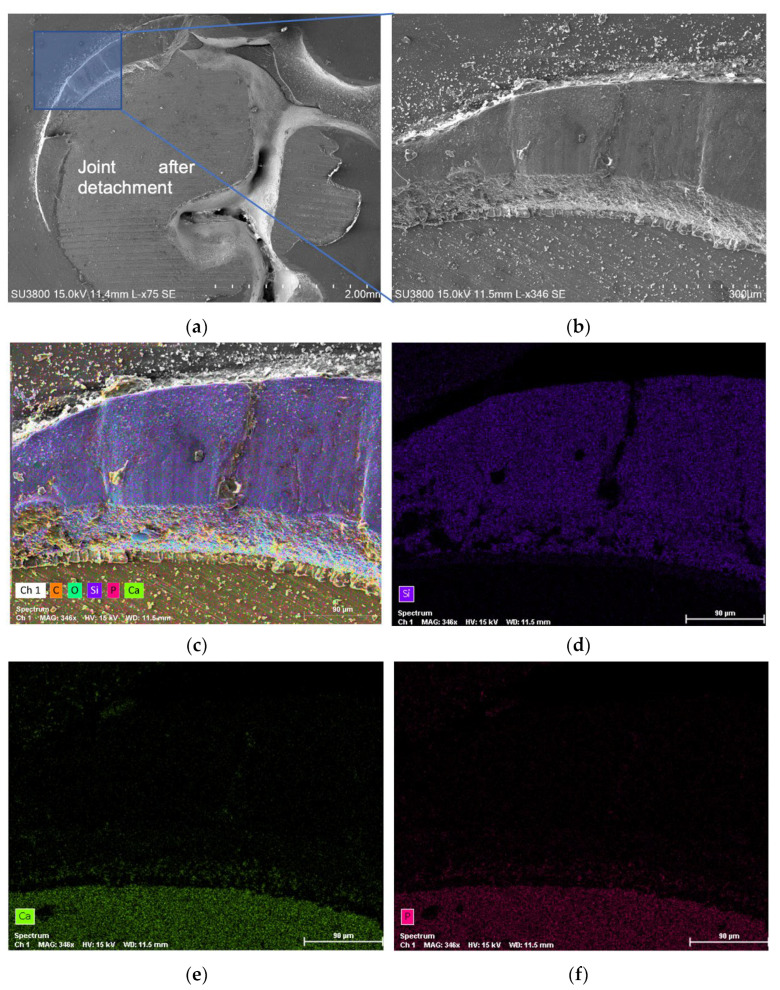
SEM micrographs and EDS analysis after the shear bond strength test, for the control group, specimen C5, showing: (**a**) joint after detachment; (**b**) part of the composite resin that was left on the edges of the joint; (**c**) spectrum map of all the chemical elements; (**d**) Si spectrum identifying the composite resin region; (**e**) Ca spectrum identifying the dentin region; (**f**) P spectrum identifying the dentin region.

Figure 6 illustrates, for the control group, SEM (scanning electron microscopy) micrographs showing the joint region after detachment (Figure 6a,b) and EDS (energy dispersive spectroscopy) analysis (Figure 6c–f) showing the spectrum map distribution of all chemical elements that were present in the surface. By observing Figure 6, it is possible to perceive that part of the composite resin was left on the edges of the joint. This same observation was reinforced by an EDS analysis, which showed a predominance of Si element (Figure 6d), characteristic of the composite resin material at the edges of the joint, and a predominance of Ca and *p* elements (Figure 6e,f) in the central region, which make up the natural mineral hydroxyapatite (dentin).

**Figure 7:**
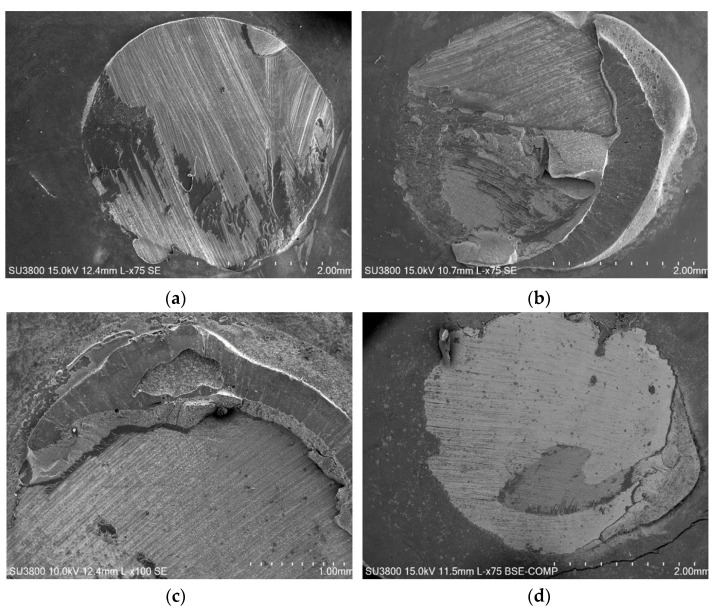

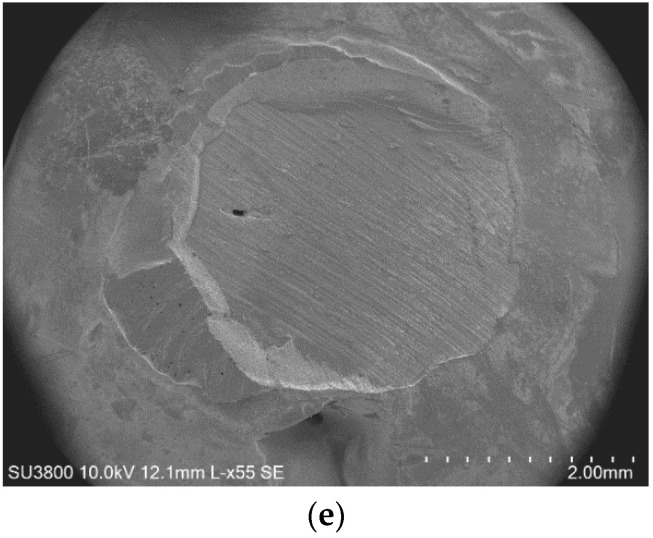
SEM micrographs, after shear bond strength test, showing joint after detachment for (**a**) Aloe vera; (**b**) chlorhexidine; (**c**) Gluma^®^; (**d**) EDTA; (**e**) ethanol.

This type of fracture mechanism, which was observed in the control samples and in all the others, constitutes a mixed fracture mode since the fracture line did not occur exclusively in the plane that divides the dentin and the composite resin (adhesive failure), being visible, mainly in the edges, parts of composite resin that remained in the glued joint. Figure 7 shows SEM micrographs at the joint region, after detachment, for all the other groups (Aloe vera, chlorhexidine, Gluma^®^, EDTA, and ethanol).

**Figure 8:**
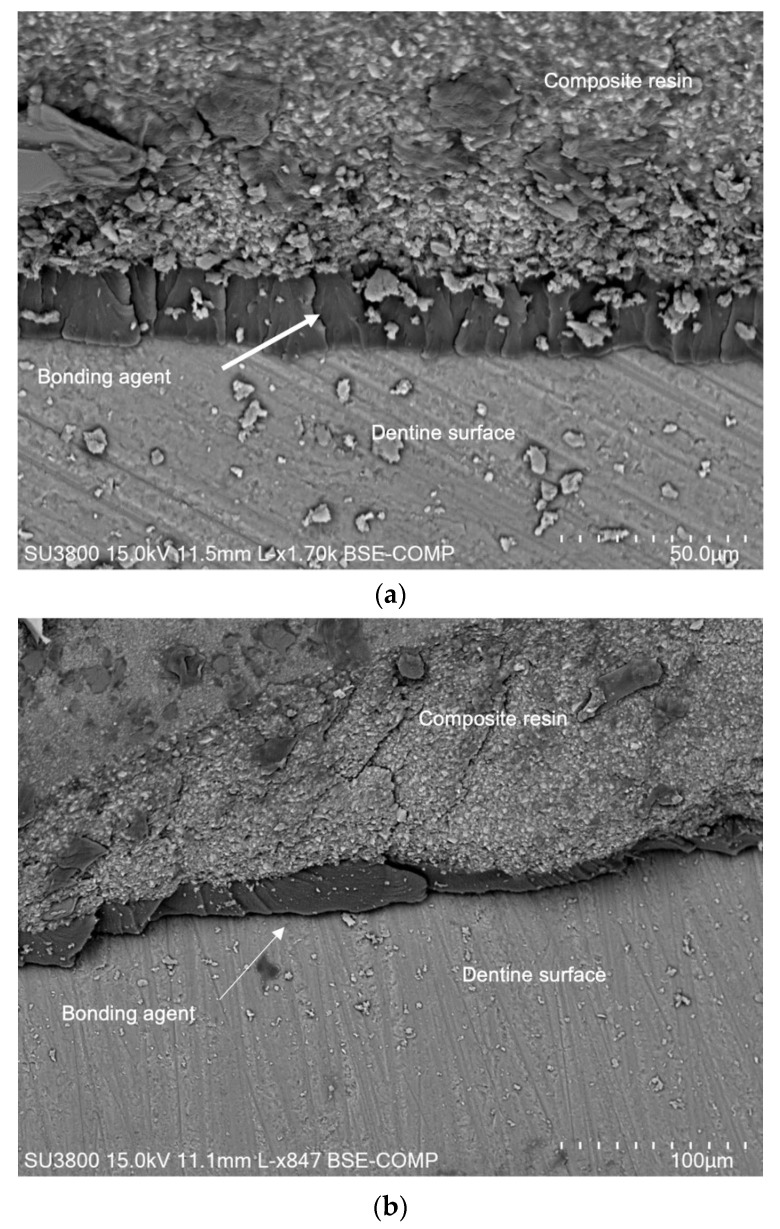
SEM micrographs after shear bond strength test, showing the bonding agent at the interface between the dentin surface and part of the composite resin that stuck to the surface for (**a**) control, specimen C5; (**b**) EDTA, specimen EDTA7.

Figure 8a shows the interface between the dentin surface and the composite resin for the control group, where it was possible to observe the adhesive joint that was composed by the bonding agent with a lamellar structure with approximately 10 μm thickness. Figure 8b shows the interface for EDTA, where it was possible to observe similar adhesive interfaces.

## 4. Discussion

A cavity disinfectant’s effect depends on each disinfectant’s characteristics, as well as on the type of substrate and materials used for the restorative procedure. Besides its biocompatibility and antibacterial action, each disinfectant should also be able to effectively disinfect the cavity without affecting bond strength values and the dentin–resin bonding interface [13,14].

Substrate-wise, when compared to enamel, dentinal tissues are completely different regarding structure and composition [15]. Dentin is a moist substrate, with a high percentage of organic components, which makes adhesive procedures much more challenging in this kind of substrate [16]. Additionally, bond strength values tend to differ according to the type of dentin, depending on whether it is healthy or caries-affected dentin and superficial or deep dentin [17,18,19]. Superficial dentin presents a higher percentage of intertubular dentin and organic components (collagen) and a lower number of dentinal tubules, which is the exact opposite of deep dentin’s composition [18,19]. Given that, superficial dentin is a much more hydrophobic substrate than deep dentin, and, as such, cavity disinfectants are much more effective in this substrate [20,21,22]. As for differences between healthy and caries-affected dentin, the latter presents not only a partially demineralized intertubular dentin, making it more porous, but also an obstruction in the intertubular area. Dental adhesives are ineffective when trying to penetrate so deeply into this kind of demineralized structure, therefore resulting in a compromised adhesive interface in this kind of caries-affected substrate [23,24].

Given the variability in substrates and in order to maximize the experimental protocol, the dentin substrate used in the present study to conduct all procedures was superficial healthy dentin, which is in accordance with the ISO/TS 11405:2015 (Dental materials—testing of adhesion to tooth structure) [12], which provides guidance on substrate selection as well as on handling and storage of samples prior to these kinds of experimental adhesive procedures. All dentinal substrates were selected from third molars and, after such extractions, all teeth were stored in distilled water until the experimental procedures. This is also in accordance with the previous ISO [12], which states that this kind of study should ideally be conducted in third molars and that, in the event that the extracted teeth are not immediately used, they should be kept in distilled water or in a 0.5% chloramine solution. No other chemical agent should be used as a storage medium, under the risk of altering the chemical and physical properties of the dentinal substrates. For the same reason, the samples’ storage should not exceed a 6-month period [12] which is why, in the present study, the teeth were only stored for 1 month until the beginning of the experimental procedures. After the completion of the adhesive and restorative procedures, all samples were stored in water at 23 °C (ISO 3696:1987, grade 3 [25]).

The present in vitro study aimed to evaluate the effect of cavity disinfectants (chlorhexidine, Aloe vera, Gluma^®^, EDTA, and ethanol) on the adhesion of the composite resin to the dentin of permanent teeth. Adhesion was not impaired by any of the studied cavity disinfectants. In fact, all groups showed higher shear bond strengths when compared to the control group, with significant differences between the control and chlorhexidine, ethanol, and EDTA groups.

After the evaluation of the samples, it was possible to conclude that the group where pretreatment with chlorhexidine was performed presented statistically significant results when compared to the control group. These results may be due to the fact that chlorhexidine has the ability to inhibit enzymes—matrix metalloproteinases (MMP)—responsible for the degradation of the collagen matrix and, consequently, the destruction of adhesive interfaces [26,27,28]. In addition, it is known that chlorhexidine has a significant antibacterial activity, as it contains gluconate, which binds to amino acids present in dentin substrates and provides this antimicrobial action [29]. It is especially effective against Gram-positive bacteria such as *S. mutans*, which is considered to be the main etiological factor of dental caries [16]. The results from the existing in vitro studies [7,15,16,18,27,29,30,31] regarding the effects of chlorhexidine on bond strength are mainly positive and in line with our research, regardless of product concentration and restorative materials, making chlorhexidine a safe option for cavity disinfection.

Regarding ethanol, although there is limited information about its use as a cavity disinfectant, the positive results of the present study are in line with the literature [15,29,32,33]. Ethanol has the capacity to expel water from dentin while keeping the collagen network expanded, which can justify the results since it provides a good substrate for composite adhesion [9,10].

EDTA has the capacity to inhibit MMP, which can relate to an increase in the longevity of the adhesive interface when it is used as a pretreatment agent. As a chelating agent, it also selectively dissolves hydroxyapatite while preventing collagen denaturation [9].

In the present study, a flowable gel-type EDTA solution containing 10% carbamide peroxide was used. Carbamide peroxide has significant antibacterial activity (including against *S. mutans* and *Lactobacillus* spp.) due to its degradation into ammonia and free oxygen species [34,35]. Although the authors were unable to identify articles that evaluated the use of carbamide peroxide as a cavity disinfectant, the results are similar to the majority of the studies [36,37,38,39] on EDTA.

Only two studies [40,41] aimed at evaluating the effect of Aloe vera as a cavity disinfectant were identified and the authors reported similar results to the ones of the present study. Aloe vera has been increasingly used in several health fields, given its antifungal, antiviral, and antibacterial (including against *S. mutans*) effects [42,43]. In addition, the ability to inhibit MMP was described [44], which may justify the results.

Even though the results were not statistically significant regarding the glutaraldehyde test group, it showed a higher shear bond strength when compared to the control group. The results revealed that adhesion was not impaired, which is in line with the available literature [7,23]. As so, glutaraldehyde may be a promising alternative as a cavity disinfection method since it has antibacterial properties and it is also capable of inhibiting the activity of endogenous MMP [45]. However, only a few published articles [7,45] on this disinfectant were identified and more studies should be conducted in order to clarify its effect on bond strength.

Considering that cavity disinfectants can be used in deep cavities, diffusion through dentin may occur, similarly to what is observed with resin monomers [46,47]. As so, further studies should evaluate the biological effect of these compounds on cell cultures, namely dental pulp cells. Additionally, studies in bacterial cultures can help to elucidate their antibacterial effect and strain specificity.

Even though this is an in vitro study and does not allow us to accurately consider the complexity of the intraoral environment [15], the development of this type of study is essential in order to strengthen scientific evidence on this matter as well as to provide a solid background to the development of clinical studies, which are scarce.

## 5. Conclusions

The use of chlorhexidine, ethanol, EDTA, glutaraldehyde, and Aloe vera as cavity disinfectants did not impair the adhesion established between the dentin of permanent teeth and composite resin in vitro. In fact, all groups showed higher shear bond strengths when compared to the control group, with significant differences between the control and chlorhexidine, ethanol, and EDTA groups.

Considering the positive results, and even though there is a need for clinical studies to support these findings, all disinfectants seem to be good choices as pretreatment agents.

## Figures and Tables

**Table 1:** Cavity disinfectants used in the study.

Material	Composition	Lot	Manufacturer
Chlorhexidine	Water, glycerin, propylene glycol, 0.20% chlorhexidine digluconate, flavor (aroma), lactic acid, PEG-40 hydrogenated castor oil, potassium acesulfame, RED 40 (CI 16035), sodium benzoate	G0007	Pierre Fabre, France
*Aloe vera*	*Aloe barbadensis* powder 200:1	OABP101	Herbs and Crops Overseas, India
EDTA	17% EDTA, 10% carbamide peroxyde, glycerin, propylene glycol	048148	Laboratorios Clarben, Spain
Ethanol	100% Ethanol	493511	Sigma-Aldrich Corporation, USA
Glutaraldehyde	35% 2-hydroxyethyl methacrylate (HEMA); 5% Glutaraldehyde	K010536	Heraeus, Germany

**Table 2:** Effect of cavity disinfectants on shear bond strength, shear modulus, and work to detachment (mean ± standard error of the mean).

Group	Shear Bond Strength (MPa)	Shear Modulus (kPa)	Work to Detachment (J/m^2^)
Control	8.34 ± 0.85	216.11 ± 19.65	4798.48 ± 990,19
Chlorhexidine	14.91 ± 1.36	189.64 ± 17.37	9347.06 ± 1603.92
Ethanol	14.45 ± 1.51	184.49 ± 20.14	8610.25 ± 2042.18
EDTA	13.30 ± 0.57	169.14 ± 18.16	6422.11 ± 827.59
Gluma^®^	11.34 ± 0.76	372.48 ± 22.31	7648.73 ± 885.66
Aloe vera	10.42 ± 0.62	317.50 ± 43.81	2283.63 ± 544.55

## Data Availability

Not applicable.
